# The Raccoon (*Procyon lotor*) as a Neozoon in Europe

**DOI:** 10.3390/ani13020273

**Published:** 2023-01-13

**Authors:** Matthias Bernhard Stope

**Affiliations:** Department of Gynecology and Gynecological Oncology, University Hospital Bonn, Venusberg-Campus 1, 53127 Bonn, Germany; matthias.stope@ukbonn.de; Tel.: +49-228-287-11361

**Keywords:** invasive species, wildlife biology, wildlife management, veterinary science

## Abstract

**Simple Summary:**

With the advance of modern civilization, there has been intensive contact among large parts of the world. This has not only encouraged increased travel and trade, but has also led to the anthropogenic introduction of animal and plant species into new areas of settlement. Such neozoa (invasive species) encounter different and sometimes completely new environmental conditions, which requires the adaptation of the neozoa to their new habitat. Under certain circumstances, the neozoa may even have significant survival advantages, for example, if the prey–hunter constellation shifts in their favor. The North American raccoon (*Procyon lotor*) was introduced into Europe by intentional and accidental releases in the 20th century and spread throughout most of Europe in the following decades. The first release was in Germany, in Central Europe, which is still the distribution hotspot of the European raccoon. Over time, raccoons were released into the wild for hunting and subsequently spread. There have also been repeated unintentional releases of raccoons by breeders, fur farms, and private pet owners. These releases have accelerated the spread of the raccoon in Europe. The influence of the raccoon on the native fauna and flora has been insufficiently studied so far, but it seems to be only marginal according to current knowledge.

**Abstract:**

The raccoon (*Procyon lotor*) is a North American half-bear that is present in much of Europe and Asia as a result of both accidental and planned releases. In Europe, raccoons were introduced primarily as a source of fur for the fur industry. In the 1930s, raccoons were released into the wild in Central Europe. At the same time, animals from fur farms and private holdings continued to enter the wild. In the following decades, the raccoon spread over large parts of Europe. In addition to the invasive spread of the Central European initial population, individual releases of raccoons occurred frequently, mainly in Southern Europe. The high adaptability of the raccoon favors its expansion into new habitats. It has a high reproductive rate, is very mobile, and encounters few predators in Europe. Raccoons have recently become a topic of interest when large raccoon populations have colonized suburban and urban areas. Despite the proximity of raccoons and humans, however, there have been hardly any conflicts to date, unlike in North America. A significant negative impact on the native fauna has been suspected but not proven. Raccoons have been identified as vectors of zoonotic diseases. Nevertheless, monitoring of the increasing numbers of raccoons in Europe seems advisable.

## 1. Introduction

The raccoon (*Procyon lotor*) is a member of the half-bear predator group (*Procyonidae*) native to North America that now occurs as a neozoon through artificial dispersal in other parts of the world [1,2,3]. In addition to the raccoon (*Procyon*), the *Procyonidae* include coatis (*Nasua*), ringtails (*Bassariscus*), and olingos (*Bassaricyon*). The raccoons include the North American raccoon (*Procyon lotor*), the most common representative of this group, as well as the crab-eating raccoon (*Procyon cancrivorus*) and the Cozumel raccoon (*Procyon pygmaeus*) [4]. Hereinafter, only the species *Procyon lotor* is discussed and is referred to as ‘raccoon’ in the text.

Adult raccoons reach a body length of up to 60 cm and a body weight of up to 12 kg. The body markings are gray to black, rarely red or brown, and occur independently of the geographical location of the habitat [5]. The body volume and body weight of the animals increases towards the northern ranges. This is in accordance with Bergmann’s animal geographic rule, where the average body size of animals increases towards the poles [4].

Raccoons are nocturnal omnivores and generalists. They occur in deciduous and mixed forests, mostly near water bodies. The population is usually larger near agricultural land or settlement areas [6]. Raccoons have also settled in suburban and urban habitats for decades [2,6,7,8]. Their diet includes plants and invertebrates, and the composition of the diet depends on the habitat and season [9]. In anthropogenically influenced habitats, a large proportion of the diet is provided by human sources [10,11]. The distribution of food resources influences the distribution of raccoons. In urban habitats, the increased supply of food and retreat opportunities leads to reduced territory sizes with higher population densities. The area of the territory is up to 702 ha in forests, up to 300 ha in rural habitats, and up to 79 ha in urban areas [11,12,13]. The population density is up to eight individuals/km^2^ in forest areas and five individuals/km^2^ in rural areas and increases significantly in urban areas to up to 333 individuals/km^2^ [8,14].

Raccoons have varying but comparatively high reproductive rates, depending on the habitat and the resources that are available in it. The mating season begins at the beginning of the year and can last until August. If a female does not become pregnant early in the year or lost her offspring, she may become pregnant again in the second half of the year [4,15]. After a gestation period of approximately 60 days, an average of five pups are born [4,15]. Raccoons living in the wild reach an age of only a few years (average, 3.1 years), but captive animals can live up to 17 years [16,17].

## 2. The Raccoons as a Neozoon

As a result of both unintentional and planned releases of raccoons outside their natural range, they are now found in parts of Europe and Asia, including Japan. As livestock for the fur industry, raccoons were kept in European fur farms as early as the early 20th century. Since 1934, here have been both planned and accidental releases of raccoons in Germany [18,19,20,21,22,23,24,25,26]. The following decades saw the spread of raccoons throughout major parts of Europe.

In the 1970s, raccoons entered Japan as pets. They were released by some pet owners and breeders and spread rapidly to almost the entire area of Japan [1].

Some characteristics of raccoons favor their invasive colonization of new habitats. Raccoons are highly adaptable and, as omnivores, can utilize a very broad spectrum of food, including anthropogenic sources. This feature is illustrated by their colonization of suburban and urban habitats. Raccoons are very good at defending themselves and have few predators, especially in newly colonized areas. Furthermore, they have a high reproductive rate [2,8]. Finally, raccoons have complex, sometimes sex-specific social behavior, which can also promote population density and habitat colonization. Raccoon pups can survive on their own from about four months of age. Female cubs often remain with their mothers, while male offspring migrate and colonize new territories [27]. Mothers and their female offspring usually form family groups, and many female raccoons remain near their mothers throughout their lives. The animals live in a common territory and have recurrent contact at sleeping and feeding sites. Young males, on the other hand, often form so-called coalitions in distant areas. A few unrelated males live in the same area and sometimes meet at sleeping and feeding sites, but without living as closely together as the female groups. This helps male raccoons defend themselves against strange males or other predators. However, in times of deprivation, such as severe winters, or during the mating season, contact between the female and male groups occurs regularly. In addition to female groups and male coalitions, solitary raccoons also occur. This is rather rare and seems to occur mainly in land areas with low raccoon population density and high resource availability. [27,28,29,30]. Population density and the intensity of social contacts depend primarily on the available resources. If sufficient food is available, there can be considerable overlapping of the territories of two raccoon groups and the number of contacts of the animals increases accordingly [18].

Raccoons prefer deciduous forests near water as habitats. Wetlands and riparian areas provide abundant food, and as omnivores, raccoons can use nearly any available trophic resource [3,31]. The availability of burrows in the ground or in plant cover for wintering and nesting is also important. [13,32,33]. Although raccoons are highly adaptable, there are environmental factors that prevent their expansion. Raccoons do not occur in deserts and boreal forests, and they cannot colonize habitats where the ground is covered with snow for 30% of the year or longer [34,35]. No raccoons have been sighted in high alpine mountains. In parts of the former Soviet Union, however, raccoons have been recorded in subalpine and alpine habitats up to 3500 m altitude [35]. The limiting factor in these extreme habitats is the permanent availability of food.

Young male raccoons spread out and can range over many hundreds of kilometers. This migration serves to expand the habitat of the species, promotes genetic variation, and prevents local overpopulation and resource depletion [36,37,38]. It is not only the extreme habitats mentioned above that pose insurmountable obstacles. Although raccoons often expand along rivers, very broad rivers and other geographic barriers may temporarily impede expansion [3,39]. However, there is no evidence that landscape barriers have caused a permanent blockade to expansion. There is even a report of a raccoon swimming across the Rhine River in southern Germany [40]. In addition, in recent decades there have been repeated releases of individual raccoons in Europe, often by private individuals, so that natural barriers are of little importance in suitable habitats [3,34,41,42].

## 3. The Distribution of Raccoons in Europe

Raccoons released in Central Germany in 1934 and in Northern Germany in the 1940s have long been considered the source of Central European raccoon populations [18,19,20,21,22,23,24,25,26]. There is now evidence that there was also a third release center in Eastern Germany [26]. Today, many decades later, these original populations still form the distribution focus of raccoons in Central Europe. It is difficult to determine the exact number of individuals in a population. However, official German hunting statistics suggest an extremely pronounced increase in the German raccoon population. While 3000 raccoons were killed in 1995, 100,000 animals killed were recorded two decades later [33]. Another analysis of hunting statistics from Germany determined the abundance index (the number of raccoons killed per hunting license), which takes into account the hunting effort. The data also demonstrate the very strong and almost exponential increase of the Central European raccoon population. While in 1995/96 there were about 100 killed animals per hunting license, in 2015/16 there were about 3400 killed animals per hunting license [34].

The descent of the Central European raccoon populations from the original release centers in Germany can be demonstrated today by population genetic analyses. In the North American habitat of origin, 76 different haplotypes were detected in 311 animals examined. In contrast, analyses of 193 individuals from Central Europe showed a comparatively low genetic diversity within the population, with only five different haplotypes [43,44]. Population genetic analyses have also supported the hypothesis that a third founding population was released in Eastern Germany [43,45]. A similar situation is found in Southern Europe. Spanish raccoon populations can also be traced back to a few individuals, according to population genetic studies [2,3,41].

The distribution of raccoons in Europe began with the release of raccoons in Germany in the 1930s and 1940s. At the same time, there were also individual official and private releases in other European countries, such as in the Soviet Union [35]. Selective individual releases of raccoons continue to have a role in the distribution of the species in Europe [3] and occur in parallel with the distribution of existing raccoon populations. Here, a distinction is made between unintentional releases (e.g., from fur farms and wildlife gardens) and planned releases (e.g., for hunting or by private livestock owners) [34].

**Figure 1:**
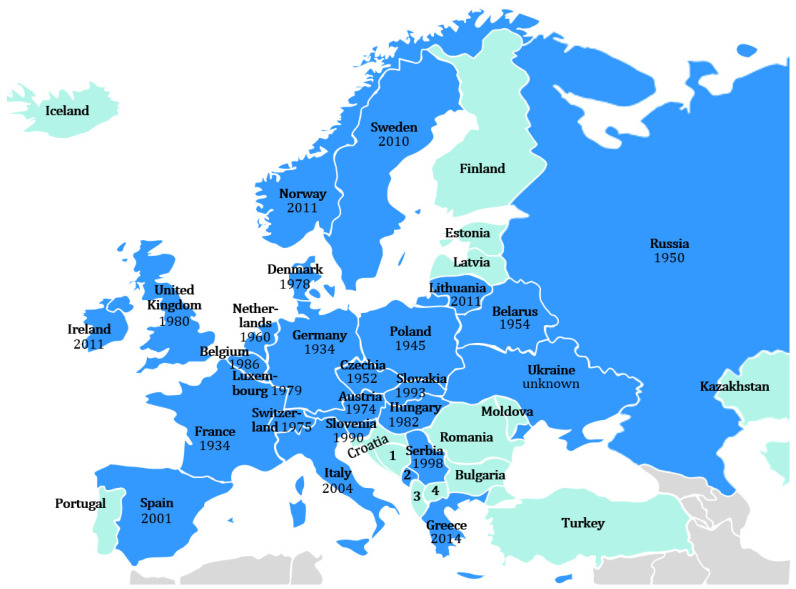
European distribution of raccoons (blue: raccoons detected, green: no raccoons detected, gray: non-European country, not considered). A state is defined as a distribution area of raccoons, regardless of the abundance and distribution of the raccoons within the state territory. The year indicates the first detection of raccoons in a national territory. Abbreviations: 1: Bosnia and Herzegovina, 2: Montenegro (1998), data collected before the independence of Montenegro and therefore no separation between Serbia and Montenegro, 3: Albania, 4: Northern Macedonia [3,31,34,35,42,46,47,48,49,50,51,52,53].

Raccoons first appeared in Germany’s neighboring countries (Denmark, Poland, Czechia, Slovakia, Austria, Switzerland, France, Belgium, Netherlands, and Luxembourg). Subsequently, they spread further to the north (United Kingdom, Ireland, Norway, and Sweden), east (Lithuania, Ukraine, Russia, and Belarus), and south and southeast of Europe (Italy, Spain, Greece, Hungary, Slovenia, and Serbia) [3,31,34,35,42,46,47,48,49,50,51,52,53] (Figure 1).

**Figure 2:**
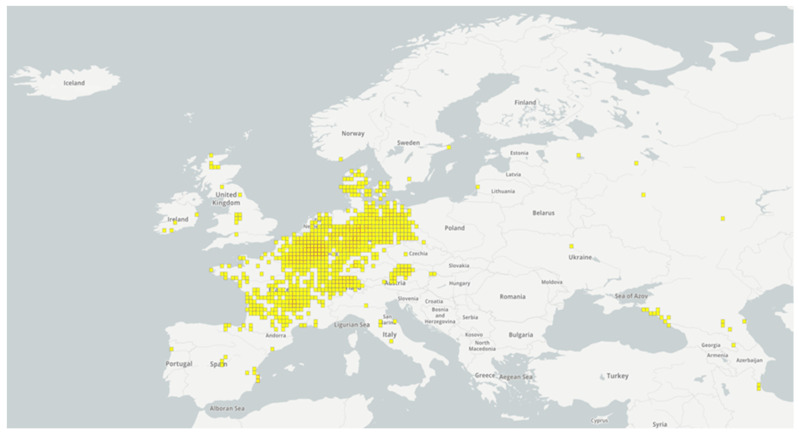
Occurrence of the raccoon in Europe. Detection of animals from 2000 to 2022 (yellow dots). Analysis and visualization of d“Procyon lotor (Linnaeus, 1758)” using the Global Biodiversity Information Facility (GBIF) in November 2022 (https://www.gbif.org/species/5218786; accessed on 18 November 2022). Basis of records: observation, machine observation, human observation, material sample, material citation, and preserved specimen.

In Central Europe, the original populations have increased and spread widely during the past nearly 100 years (Figure 2), mainly in Germany, Denmark, the Netherlands, Belgium, Luxembourg, and France. As a result, there are large areas with raccoon populations that are in contact with each other depending on geographic conditions. In Southern Europe (Southern France, Spain, and Italy), in contrast, there have been repeated individual releases [3,34,41,42], as well as in France very early in the last century. Due to this patchy release, raccoons already occur very widely in Southern Europe, although the main distribution area is far away. Thus, the animals often live in geographically separated populations. A similar situation can be found in Eastern and Southeastern Europe. Here, too, only isolated, geographically separated raccoon populations are found. In the literature, there are single records of animals that have not been deposited in the Global Biodiversity Information Facility (GBIF) database. It is therefore possible that some of the areas shown have not been permanently inhabited by the raccoon [34].

A special situation is found in the raccoon populations on the territory of the former Soviet Union. In the 20th century, raccoons were released into the wild on a large scale in a number of Soviet republics with the support of the government and with scientific assistance [35]. The animals originated from zoological gardens of the Soviet Union or, in a few cases, from raccoon farms in Western Europe. In addition, raccoons living in the wild were caught in live traps and released in other areas. Raccoons were released in Central Asia (Kyrgyzstan) as early as 1936. Further releases followed in the Far East (Asian part of Russia 1937), Eurasia (Azerbaijan 1941), Southern Russia (1950), and Central Asia (Uzbekistan 1953). In some cases, raccoons were released several times in one area or in closely located areas. From 1936 to 1958, a total of 1253 individuals were released throughout the former Soviet Union [35,54,55].

**Figure 3:**
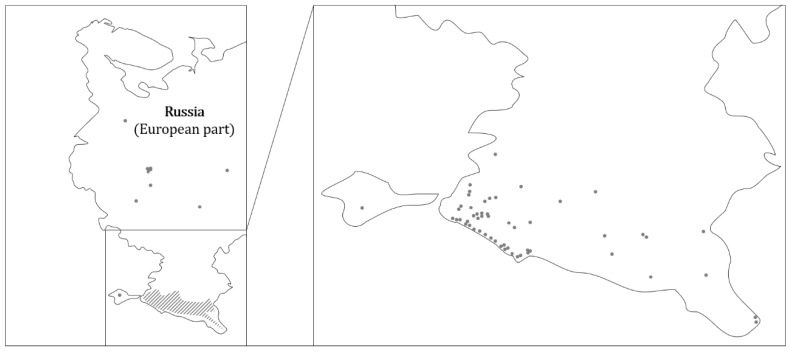
Occurrence of the raccoon in the European part of Russia (dark grey dots). Raccoons are repeatedly spotted in European Russia (left). However, the main populations are located in the Caucasus and on the Black Sea coast of the extreme southern part of European Russia (right). Distribution data were obtained from the Mammals of Russia portal (https://rusmam.ru/atlas/map; accessed on 18 November 2022)) in November 2022.

Currently, raccoons are frequently found in the European part of Russia, but there are no systematic studies on this issue. The Mammals of Russia portal (https://rusmam.ru/atlas/map; accessed on 18 November 2022) shows habitats in the more temperate climatic zones of Russia (Figure 3), e.g., at Lake Onega in the north, in the greater Moscow area, and in the east in the area of the city of Kazan. The main raccoon population is found on the southern edge of European Russia. Raccoons live along the Black Sea coast and throughout the Caucasus to the Caspian Sea. Counts from 1964 estimated about 25,000 animals in this region [35]. The high population density may be due to the fact that the Caucasus was a focal point of raccoon settlement in the former Soviet Union during the last century [36].

Many neozoa have been intentionally or unintentionally introduced into the former Soviet Union. The potential ecological impacts of these neozoa on the environment have been tracked for a long time [56]. In the case of the raccoon, there is evidence that other species, e.g., ground-nesting birds, reptiles, and amphibians, have been predated or displaced regionally and seasonally. This has also been shown in studies in the rest of Europe. Other predators, such as the European mink (*Mustela lutreola*), may be displaced from their habitats by raccoons [2,57,58,59].

## 4. The Impact of Raccoons on European Habitats

One interaction of raccoons with their environment that can be particularly well perceived by humans is cohabitation in the same habitat and resulting contacts. In North America, raccoons are considered pests. The animals cause crop damage and can attack domestic and farm animals. Furthermore, damage and soiling of homes, equipment, and other facilities occurs with corresponding economic impacts [60,61,62]. A similar situation could be suspected of Europe, but there are hardly any systematic studies on this question. The city of Kassel (Germany), located in Central Europe, is one of the oldest hotspots of raccoon release in Europe. Accordingly, populations have been established in the city area and its surroundings. The city is believed to have the highest raccoon density of all European cities [63]. In towns, raccoons have a much lower escape distance from humans and other large animals. Together with the extensive supply of food and shelter, this makes the high density of raccoons possible. Nevertheless, wild raccoons living in suburban and urban habitats went unnoticed for a long time. It was not until the 1960s that wild raccoons were discovered in human settlements [63,64]. Even in densely populated urban centers, there are sufficient opportunities for raccoons to survive (parks, gardens, cemeteries). However, raccoon densities decrease from the urban fringe to the urban center, probably due to decreasing resources for survival [18,64]. In contrast to North America, the overall perception of urban raccoons in Europe does not appear to be solely negative. The animals are often even fed by residents in gardens or housing developments [32,63,65]. Over the decades, all attempts to control raccoon populations in urban areas have proven ineffective. Resettlement and various hunting options have ultimately been unsuccessful [18,34,63]. A very similar situation is found in other European habitats, although there is much less data available than for Central Europe [41,42,66].

Neozoa can also have impacts on the ecosystem through interaction with native animal species. Predator–prey interactions and competition for food and sleeping or nesting sites can cause ecological shifts to occur. This has been suggested for the raccoon as a medium-sized predator in Europe [31], but to date there are no robust data on this question [56,66,67,68,69]. There are no large predators in the European distribution range of raccoons that could limit the raccoon population. Comparable native predators are the European mink (*Mustela lutreola*), polecat (*Mustela putorius*), European badger (*Meles meles*), and raccoon dog (*Nyctereutes procyonoides*), which has also been released as a neozoon in Eastern Europe. Interactions of these predator species with raccoons have not been observed to date. The decrease in the European raccoon dog population is not due to competition with the raccoon, as was first suspected, but has been attributed to infection with canine distemper virus [69,70]. In North America, raccoons prey on ground- and cavity-nesting birds, especially in geographically isolated habitats, such as islands [71,72]. The ecological impact of raccoons in Europe has been poorly studied. The results of studies on the influence of raccoons on European ecosystems are difficult to interpret statistically because of their small number. Currently, the impact of raccoons on the native fauna is considered to be rather low.

## 5. Raccoons as Vectors of Zoonotic Diseases

One area of concern in interactions between neozoa and the environment is the transmission of pathogens. Because raccoons have strong populations in suburban and urban habitats, this issue affects not only other wildlife, but also livestock and humans [2]. In anthropogenic habitats, infectious diseases are among the most common causes of mortality in raccoons [8,14,73]. In extreme cases, neozoa can not only increase disease transmission, but also introduce new pathogens [74]. In their North American habitat, raccoons are intermediate and final hosts for a number of zoonotic pathogens that regularly lead to infections in humans [75,76,77,78,79]. However, a much lower spectrum of parasites and zoonotic pathogens has been detected in wild raccoons in Central Europe and Germany than in North American raccoons [80,81].

The raccoon roundworm (*Baylisascaris procyonis*) is the most common parasite of the raccoon. The raccoon is the final host of the nematode and excretes the worm eggs with its feces. These eggs, when ingested by humans, can cause serious and fatal infectious diseases. Particularly in urban habitats, a large number of nematode eggs can be released into the environment due to the high raccoon density. The eggs are extremely resistant to environmental influences and are infectious for a long time. In North America, the rate of infestation of raccoons with the raccoon roundworm is up to 80% and represents a corresponding risk of infection in humans [82,83]. In Europe, only a few cases of infectious diseases transmitted by raccoons have been described. In 1991, for example, one case was documented in which a raccoon keeper in Germany became sick due to a raccoon roundworm infection [84].

In addition, there are several other helminth species that parasitize in raccoons. Besides *Baylisascaris procyonis*, human pathogenic species are, for example, *Trichinella* species and *Strongyloides procyonis*, which has also been found in raccoons in Eastern Europe [31]. There are also raccoon pathogenic species from the protozoan group that can be transmitted to humans. These include members of the species *Trypanosoma*, *Toxoplasma*, *Cryptosporidium*, *Neospora*, and *Sarcocystis*, which have been detected sporadically in European raccoons [2,81,85].

Among the most important bacterial zoonotic pathogens in raccoons are two species, *Leptospira* and *Salmonella*. Bacteria of these groups are considered hazardous zoonotic pathogens worldwide, affecting a wide range of host animals [86,87]. In North America, a number of other bacterial species have been identified in raccoons, including *Francisella*, *Mycobacterium*, *Borrelia*, and *Rickettsia* [2,81]. The bacterial infection status of European raccoon populations has rarely been systematically studied. However, the few random analyses available suggest that the broad bacterial spectrum and sometimes high infestation of North American raccoons have not been transferred to the new habitats in Europe [81,88,89].

Viral pathogens in raccoons include the rabies virus. Rabies is a fatal and incurable zoonotic encephalitis that can affect almost all carnivores [90,91]. Often, the various animal pathogenic virus types can infect other animal species. In Europe, canine rabies is considered largely eliminated. However, rabies reservoirs exist in wild animals, for example, in foxes, raccoon dogs, and bats [92,93]. Rabies virus-positive raccoons have been reported sporadically in Central and Eastern Europe [2]. The causative agent of canine distemper, canine distemper virus, is also consistently documented in wildlife, including raccoons. Most canine distemper virus infections in raccoons are described at the end of the mating season and during the migration of the young male raccoons [94,95]. Viral strains isolated from Central and Southern European raccoons have all been assigned to the phylogenetic European group [96,97,98]. Several other viruses, such as parvoviruses, pseudorabies virus, and adenoviruses, have been detected in North American raccoon populations, but not yet in European populations [2].

Overall, the risk of infection with zoonotic pathogens from raccoons in Europe appears to be rather low. This may be due to the lower spectrum of pathogens in European raccoon populations. For risk assessment, however, the current scientific study situation in Europe must also be considered. There are fewer scientific analyses regarding the health and infection status of raccoons compared to research in North America. As is often the case in wildlife biology and wildlife medicine, this is due to the difficulty in accessing individuals in a population. In addition, the animals’ high local mobility and rapid distribution into new habitats makes systematic documentation of infestation rates challenging. Only random samples with usually low numbers of cases are available, from which extrapolations are made to populations with often high numbers of individuals, at least in Central Europe. For almost all of Europe, these surveys are not carried out systematically and over longer periods of time, but represent individual projects of single research institutions. Nevertheless, it seems advisable to monitor the potential risk of infection from wild raccoons. Systematic and, if favorable, Europe-wide programs to screen European raccoon populations for pathogens might help.

## 6. Outlook

Raccoons have been living in Europe for almost a century. Their numbers and the areas they inhabit within Europe are constantly increasing. In areas lacking in humans and close to nature, raccoons spread slowly. Here, contact with humans has a subordinate role. However, as with other predators (such as the wolf), conflicts can occur. In suburban and urban habitats, humans have become accustomed to the presence of raccoons and other urban wildlife, such as the fox, wild boar, and marten. Potential conflicts due to feeding damage or damage to facilities occur, but do not seem to prevent a predominantly consensual coexistence between humans and animals. Raccoons in densely populated urban areas have changed their behavior. They have reduced shyness and can coexist more closely with their own species. This, together with their adaptation to the wide anthropogenic food supply, enables the enormous population densities in urban habitats.

Management strategies for controlling the population size and dispersal potential of raccoons have failed so far. Hunting and wildlife biology programs, as well as relocation campaigns fail because of the high reproductive rate and the high mobility of the animals. Thus, it seems reasonable to accept raccoons as a neozoon and therefore as part of the European fauna. A feared negative impact on the overall native flora and fauna has not been observed so far, but regional studies show predation or displacement of native species. This ecological influence may depend on seasonal variation in food availability. The danger of zoonotic pathogens seems to have been overestimated. The pathogen spectrum of wild European raccoons is significantly smaller than that of North American raccoons. Accordingly, there have been few cases in Europe of zoonoses attributed to contact with raccoons. It is possible, however, that all of these effects depend on population density. Systematic monitoring of raccoon habitats, their population densities, and pathogen reservoirs therefore seems advisable. This would be all the more effective if carried out on a European level.

## 7. Conclusions

The raccoon has been spread throughout many parts of Europe by both accidental and planned releases. In this process, the raccoon’s adaptability favored its colonization and survival in new habitats. A high reproductive capacity and mobility, the absence of natural predators, and the use of a wide range of resources are the most striking characteristics of the raccoon. These characteristics even have culminated in the raccoon’s ability to colonize suburban and urban habitats with great success. Conflicts with endogenous fauna and humans are present, but are nowhere near as significant as in the raccoon’s original distribution range in North America. Programs to eradicate raccoons in parts of Europe have regularly failed. Thus, raccoons should be considered part of the European wildlife.

## Data Availability

Not applicable.
